# Supplementary material for: Targeting Periostin Expression Makes Pancreatic Cancer Spheroids More Vulnerable to Natural Killer Cells
Source: Biomedicines. 2023 Jan 19;11(2):270. doi: 10.3390/biomedicines11020270 (PMC9952976; doi:10.3390/biomedicines11020270)
Supplement: Supplementary file 1 [file biomedicines-11-00270-s001.zip › biomedicines-2060890-supplementary.pdf]

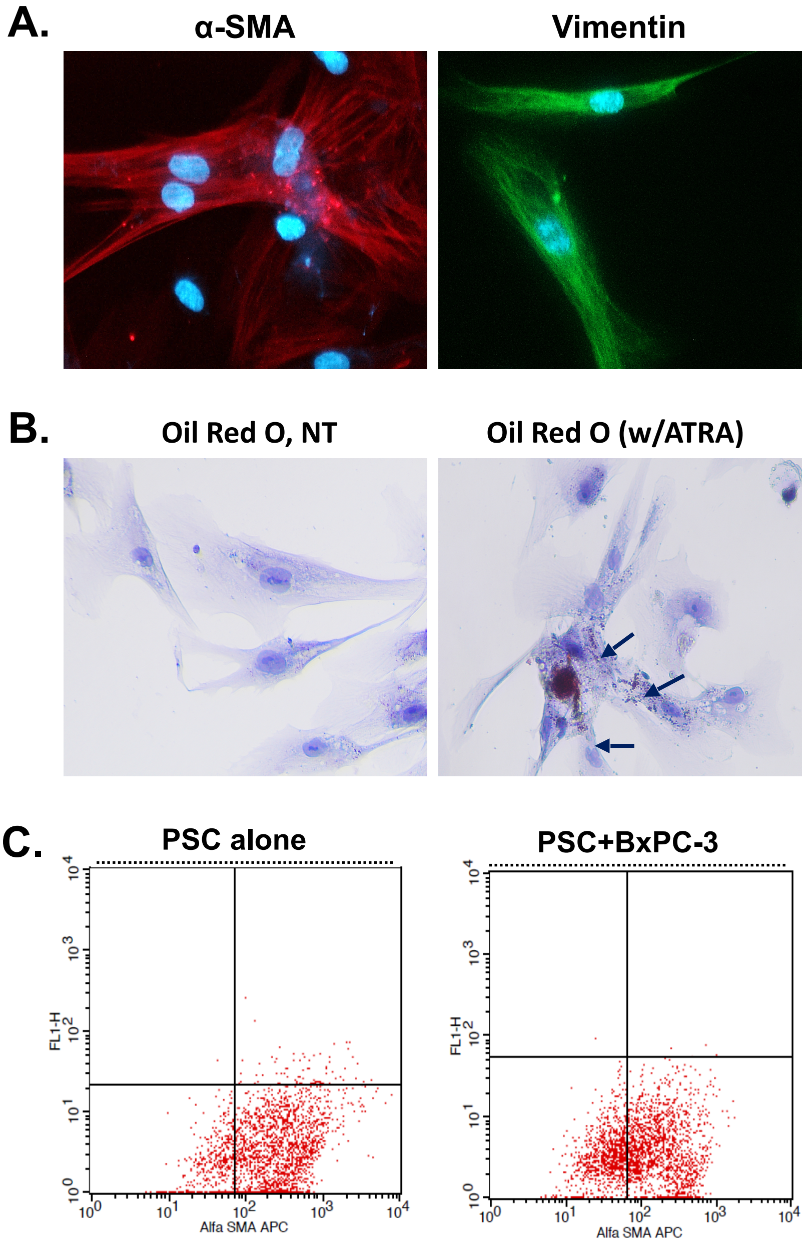

Figure S1. (A, B) The activation status of PSC cells was determined by α-SMA **(A)** and Oil Red O **(B)** staining. **(C)** α-SMA positivity was analyzed by flow cytometry in both PSC mono-culture and BxPC-3+PSC co-culture spheroids.

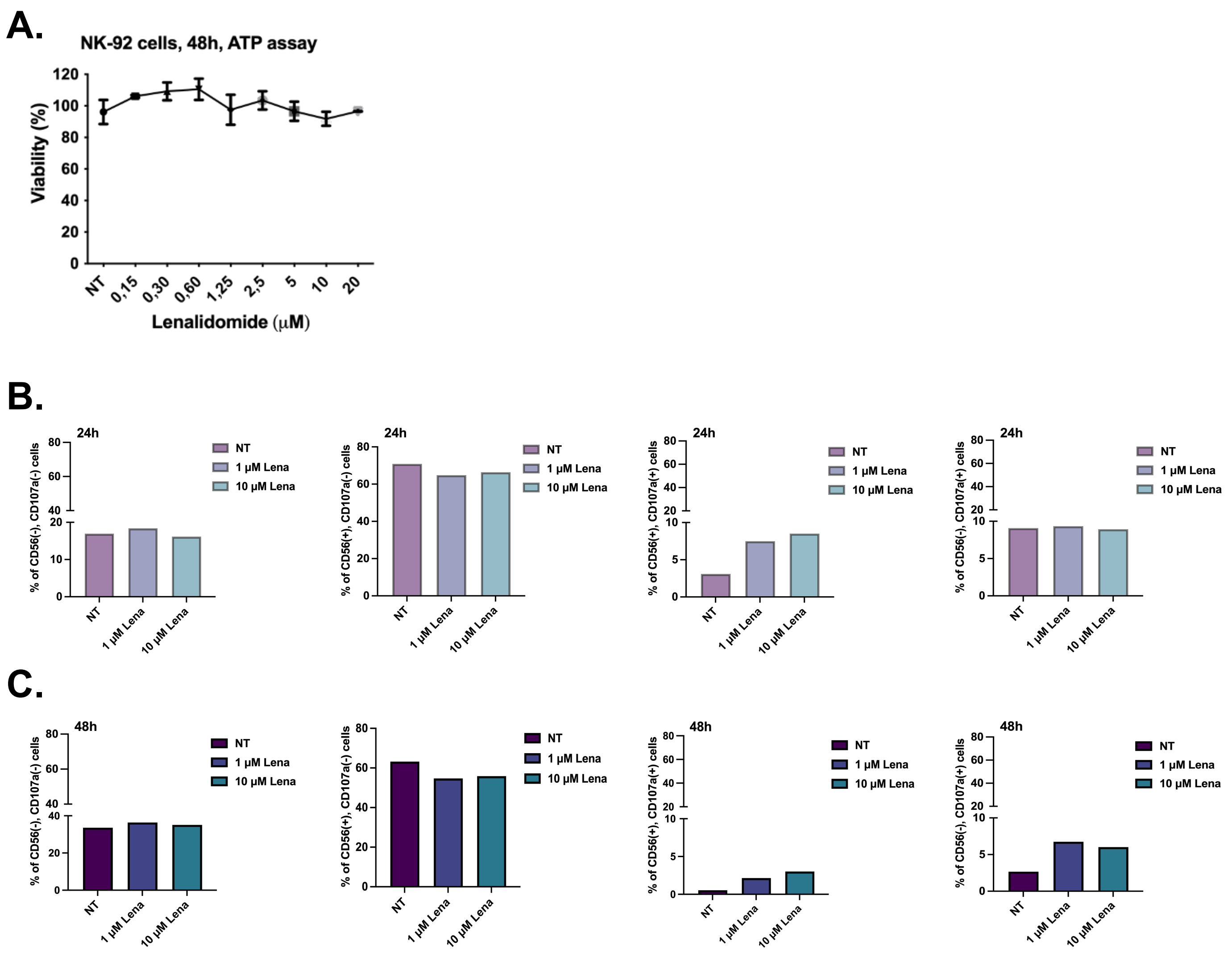

Figure S2. (A) NK-92 cells were treated with different concentrations (0.15-20 μM) of lenalidomide for 48h, and cell viability assessed by ATP assay. **(B,C)** The quantitative graphs for CD107a positivity in CD56 positive NK-92 cell population were evaluated after stimulation by either 1 or 10 μM lenalidomide. Graphs show CD107a status of the cells after **(B)** 24 h and **(C)** 48 h stimulation by lenalidomide.
